# Supplementary material for: Effect estimates can be accurately calculated with data digitally extracted from interrupted time series graphs
Source: Res Synth Methods. 2023 Jun 9;14(4):622–38. doi: 10.1002/jrsm.1646 (PMC10946754; doi:10.1002/jrsm.1646)
Supplement: Supplementary file 2 — Appendix S2. [file JRSM-14-622-s003.docx]

# Supplementary File 2

This document contains supplementary images and information for the study:

“Effect estimates can be accurately calculated with data digitally extracted from interrupted time series graphs”

Simon Lee Turner*, Elizabeth Korevaar, Miranda S Cumpston, Raju Kanukula, Andrew B Forbes, Joanne E McKenzie.

School of Public Health and Preventive Medicine, Monash University, Melbourne

* Correspondence: Simon Turner, School of Public Health and Preventive Medicine, Monash University, Melbourne, 3004, Victoria, Australia. Email: [simon.turner@monash.edu](mailto:simon.turner@monash.edu)

## Supplementary image 1: method for scaling confidence intervals


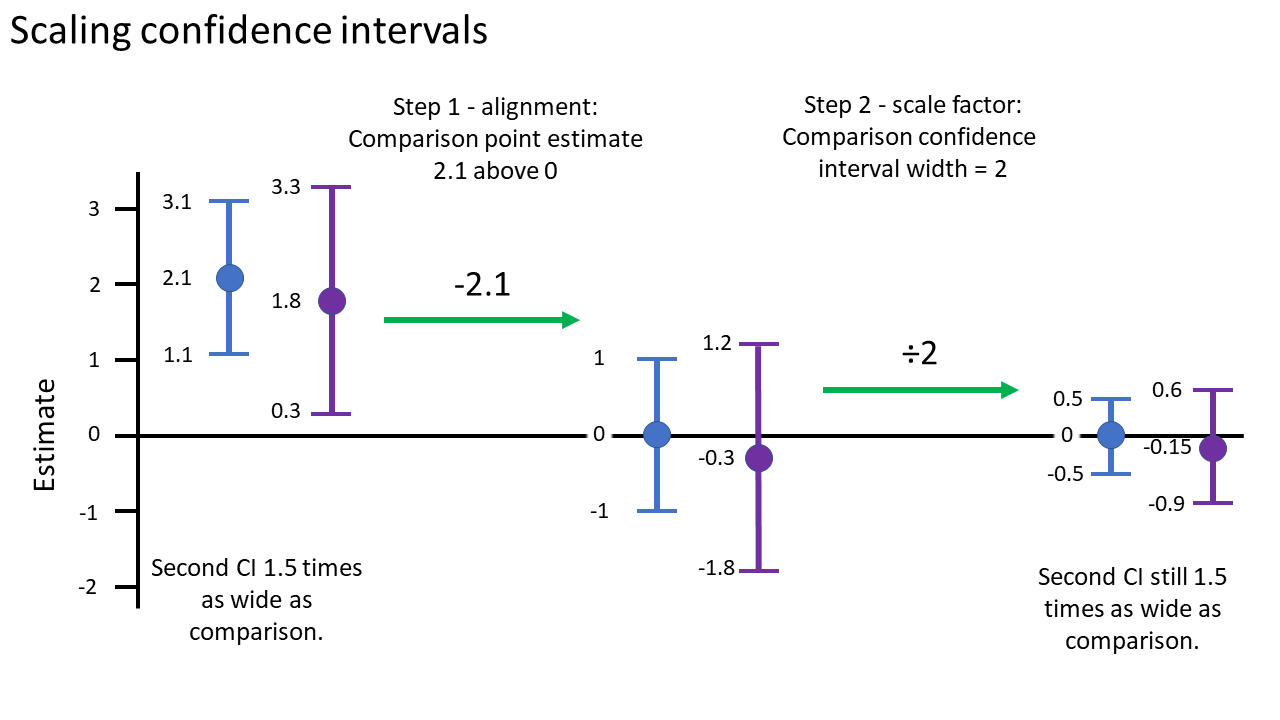


Figure S2.1: Diagram showing the two-step process for scaling confidence intervals for Figure 9 in the manuscript (described in section 2.4.3 and 3.5). In the first step, the point estimates are aligned to the y-axis so that the comparison point estimate (blue) is set to zero. This is achieved by subtracting the comparison point estimate (in this example, 2.1) from itself, its confidence limits, and the comparator (purple) point estimate and its confidence limits. In the second step, the confidence interval limits are scaled so that the comparison confidence interval limits range from -0.5 to 0.5. This is achieved by dividing the point estimates and confidence interval values by the width of the comparison confidence interval (in this example, 2). This process maintains the relative magnitudes of the difference in point estimates and confidence interval widths and allows for comparisons across datasets with different scales and results.

## Supplementary image 2: pairwise p-value comparisons for level change


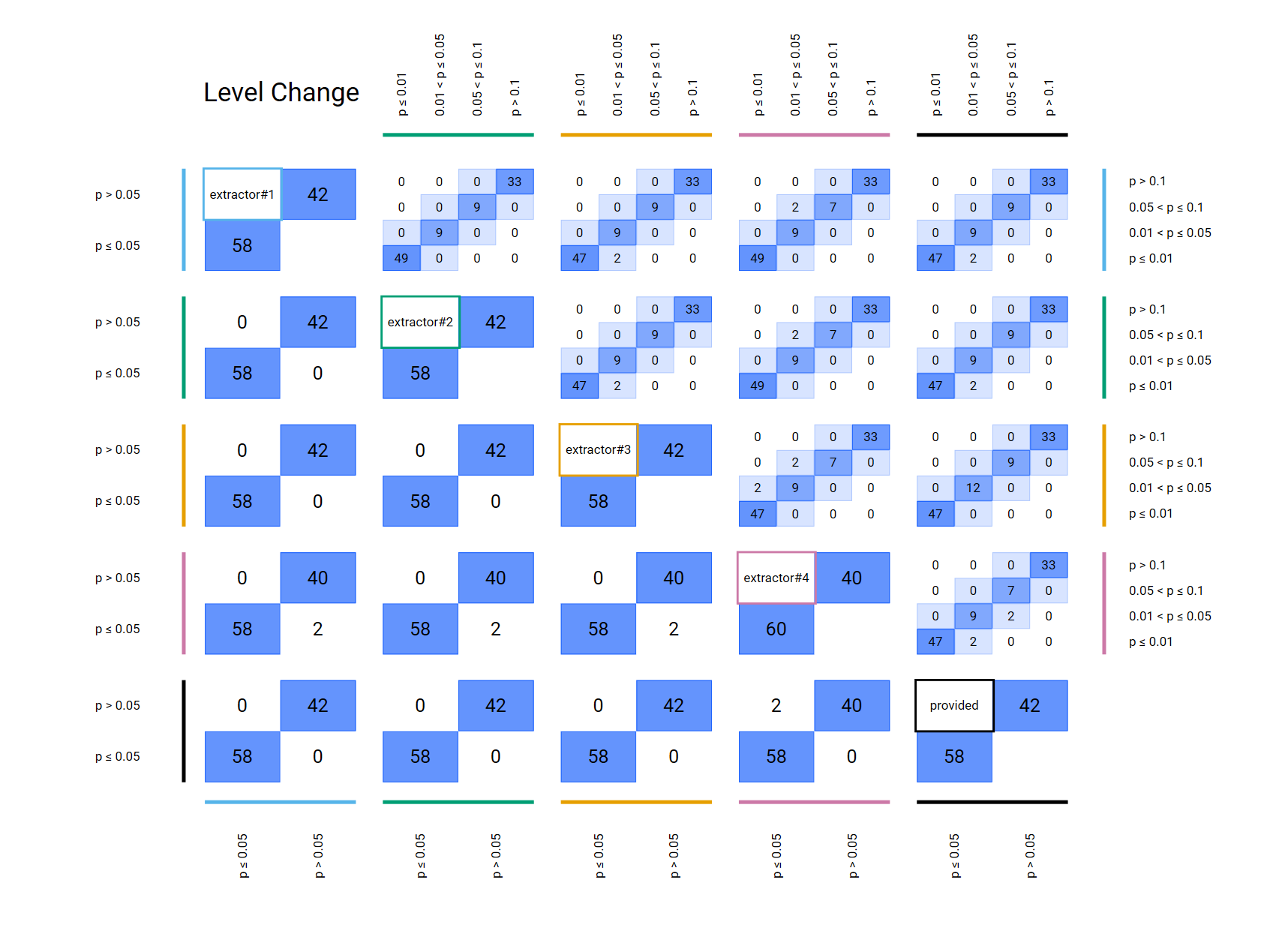


Figure S2.2: Pairwise agreement in statistical significance of estimates of p-value comparisons for level change (referred to in section 3.6 in the manuscript). In the top triangle, boxes are divided into 16 cells with p-values categorised using a fine gradation of statistical significance, namely, p-value ≤ 0.01, 0.01<p-value≤0.05, 0.05<p-value≤0.1, p-value>0.1. In the bottom triangle, boxes are divided into four cells with p-values categorised at the 5% level of statistical significance (i.e. ≤0.05, >0.05). Each cell within a box contains the percentage of effect estimate p-values falling within the row and column defined statistical significance levels. The colour bands surrounding the left/right and top/bottom side of the plot indicate the two data sources being compared. For example, within the box comparing extractor 4 and provided time series in the bottom triangle, in 2% of the time series the level change estimate from the provided time series yielded a p-value > 0.05 while that from extractor 4 yielded a p-value ≤ 0.05 (top left cell). Numbers may not add to 100 due to rounding.

## Supplementary image 3: pairwise p-value comparisons for slope change


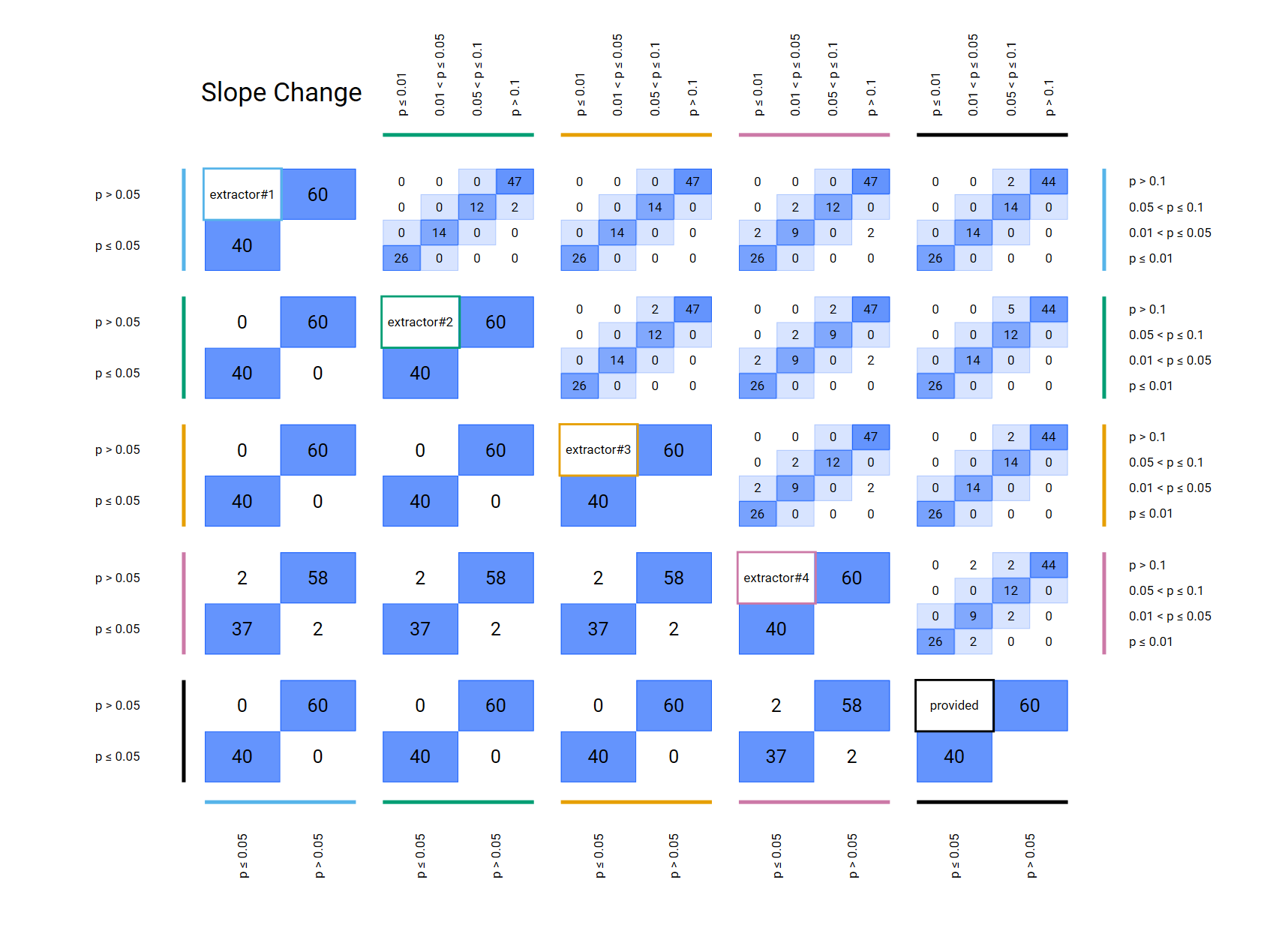


Figure S2.3: Pairwise agreement in statistical significance of estimates of p-value comparisons for slope change (referred to in section 3.6 in the manuscript) In the top triangle, boxes are divided into 16 cells with p-values categorised using a fine gradation of statistical significance, namely, p-value ≤ 0.01, 0.01<p-value≤0.05, 0.05<p-value≤0.1, p-value>0.1. In the bottom triangle, boxes are divided into four cells with p-values categorised at the 5% level of statistical significance (i.e. ≤0.05, >0.05). Each cell within a box contains the percentage of datasets falling within the row and column defined statistical significance levels. The colour bands surrounding the left/right and top/bottom side of the plot indicate the two data sources being compared. For example, within the box comparing extractor 4 and provided time series in the bottom triangle, in 2% of the time series the slope change estimate yielded a p-value > 0.05 while that from extractor 4 yielded a p-value ≤ 0.05 (top left cell). Numbers may not add to 100 due to rounding.

## Supplementary image 4: example of ITS graph missing a data point


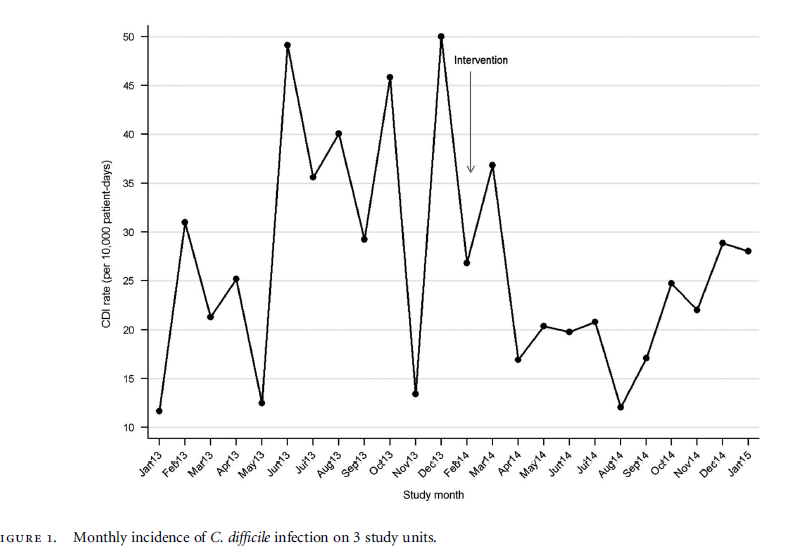


Figure S2.4: Manuscript image with no January 2014 on the x-axis, leading to 24 time points plotted, though the range of the axis would indicate there should be 25. Reproduced with permission from Cambridge University Press, license number 5376321103027, © Infection Control & Hospital Epidemiology. Figure 1, David A. Pegues, Jennifer Han, Cheryl Gilmar, Brooke McDonnell, Steven Gaynes. (2016). Impact of Ultraviolet Germicidal Irradiation for No-Touch Terminal Room Disinfection on Clostridium difficile Infection Incidence Among Hematology-Oncology Patients. Infection Control & Hospital Epidemiology), 38(1), 39-44. <https://doi.org/10.1017/ice.2016.222>

## Supplementary image 5: example of ITS graph missing a data point


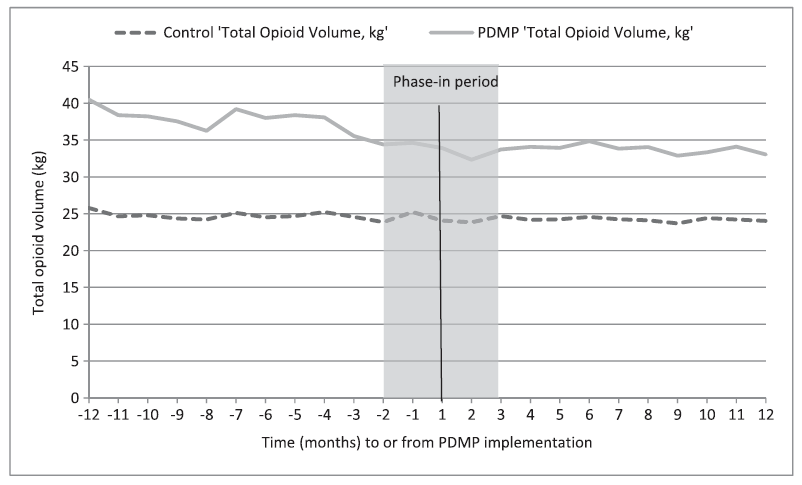


Figure S2.5: Manuscript image with no zero on the x-axis, leading to 23 time points plotted, though the range of the axis would indicate there should be 24. Reproduced with permission from Wiley, license number 5366320934137, ©Society for the Study of Addiction. Figure 1, Moyo, Patience, Simoni‐Wastila, Linda, Griffin, Beth Ann, Onukwugha, Eberechukwu, Harrington, Donna, Alexander, G. Caleb, & Palumbo, Francis. (2017). Impact of prescription drug monitoring programs (PDMPs) on opioid utilization among Medicare beneficiaries in 10 US States. Addiction (Abingdon, England), 112(10), 1784–1796. <https://doi.org/10.1111/add.13860>

## Supplementary image 6: full size version of Figure 1


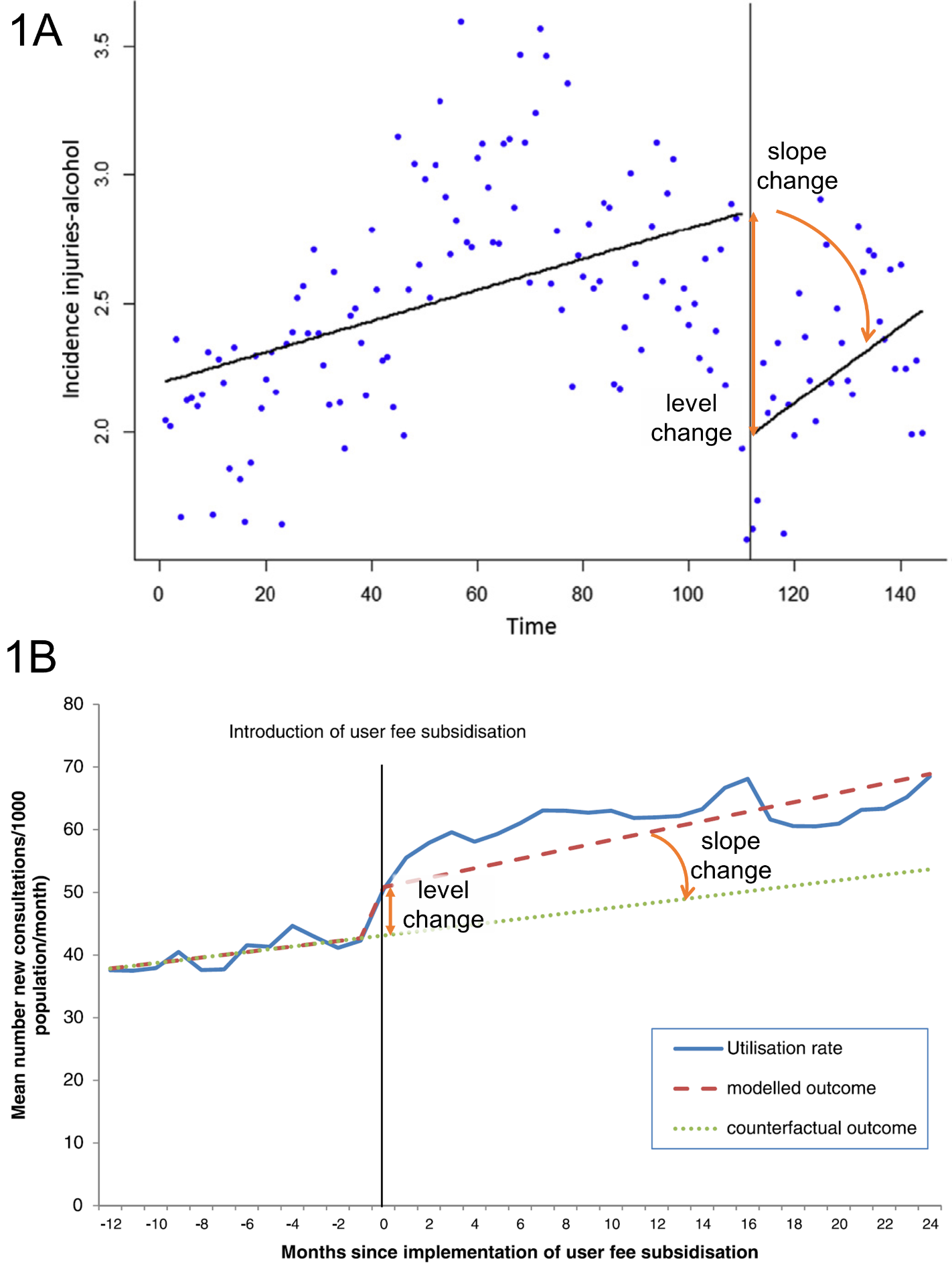


Figure S2.6: Examples of interrupted time series graphs. Figure 1A shows incidence rate of injuries related to alcohol per 100,000 inhabitants over time (months) before (left) and after (right) the implementation of a law decreasing the legal blood alcohol limit for driving in Chile, South America. Figure 1B shows the effects of user fee subsidisation on mean health-care utilisation rate 12 months prior and 24 months following their introduction for 16 health zones of the Democratic Republic of Congo (2008 to 2012). Level change and slope change labels and indications (orange arrows) have been added for clarity. Figure 1A reprinted from Public Health, 150, Nistal-Nuño B, “Segmented regression analysis of interrupted time series data to assess outcomes of a South American road traffic alcohol policy change”, 51-59, Copyright (2017), with permission from Elsevier, license number 5376221132966. Figure 1B reprinted from BMC Health Services Research, 14:504, Maini R et al., “Picking up the bill - improving health-care utilisation in the Democratic Republic of Congo through user fee subsidisation: a before and after study”, Copyright (2014), under the terms of the Creative Commons Attribution License 2.0 (http://creativecommons.org/licenses/by/2.0).

## Supplementary image 7: full size version of Figure 2


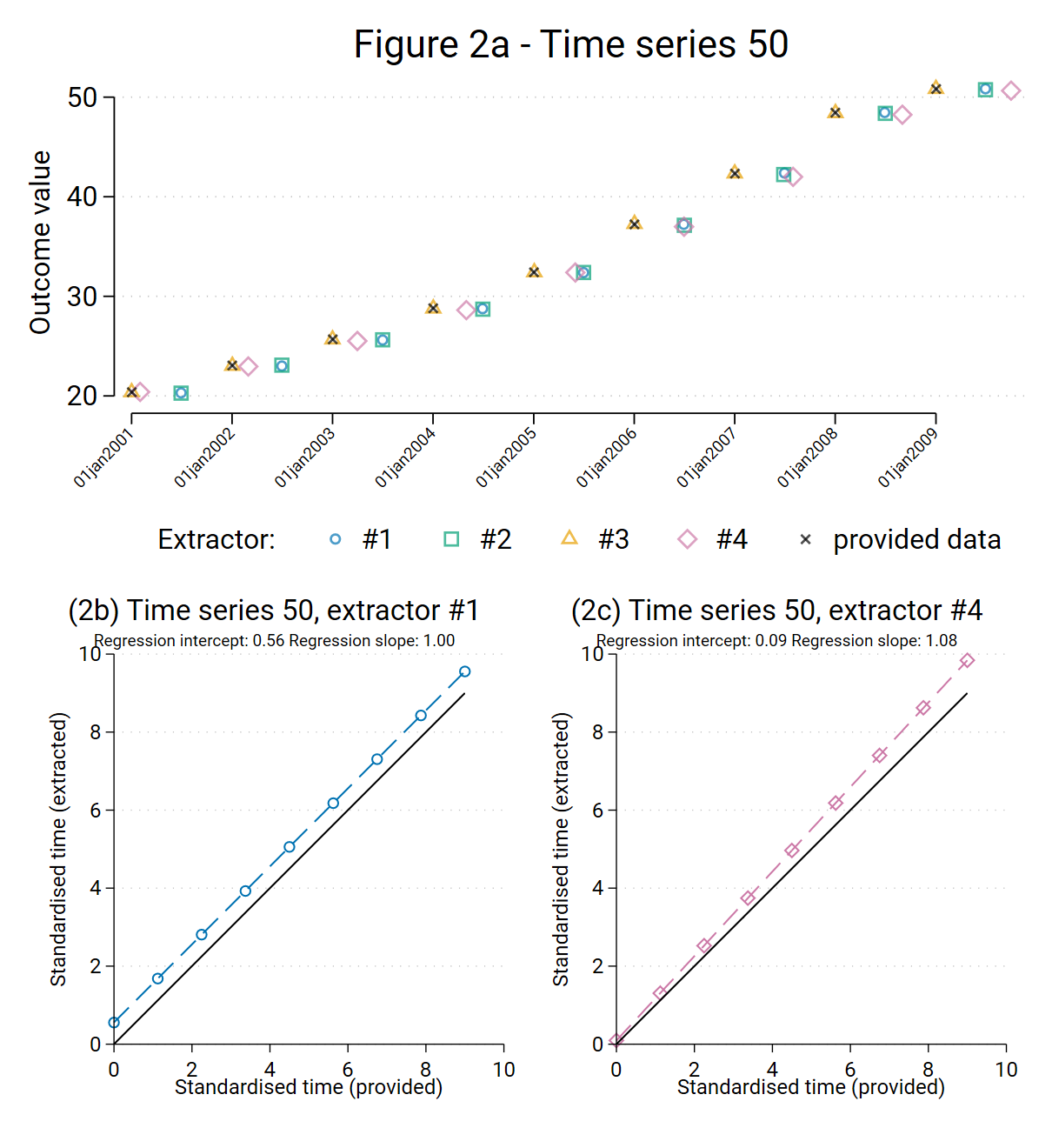


Figure S2.7: Example demonstrating how different types of errors in the extraction of time points (x-coordinates) from time series plots can be detected via linear regression.
(2a) Scatterplot of provided (black x symbol) and extracted data from four extractors (represented by different coloured symbols). The blue circle (and green square) represents extracted data points that are consistently half of a time unit greater than the provided data. The purple diamond represents extracted data that is close to the correct time at the start of the series, but with increasingly large error over the series (see (2c) for how linear regression can be used to quantify this error). Figures 2b and 2c demonstrate how linear regression can be used to quantify these errors.
(2b) Scatterplot of x-coordinates extracted by one extractor (#1 indicated by blue circles) against the provided data. The extracted data is consistently half of a time unit greater than the provided data. This is reflected in the regression intercept estimate of 0.56.
(2c) Scatterplot of x-coordinates extracted by one extractor (#4 indicated by purple diamonds) against the provided data. The extracted data, which is close to the provided data at the start of the series, has increasingly larger error over the series. This is reflected in the regression slope estimate of 1.08.

## Supplementary image 8: full size version of Figure 3


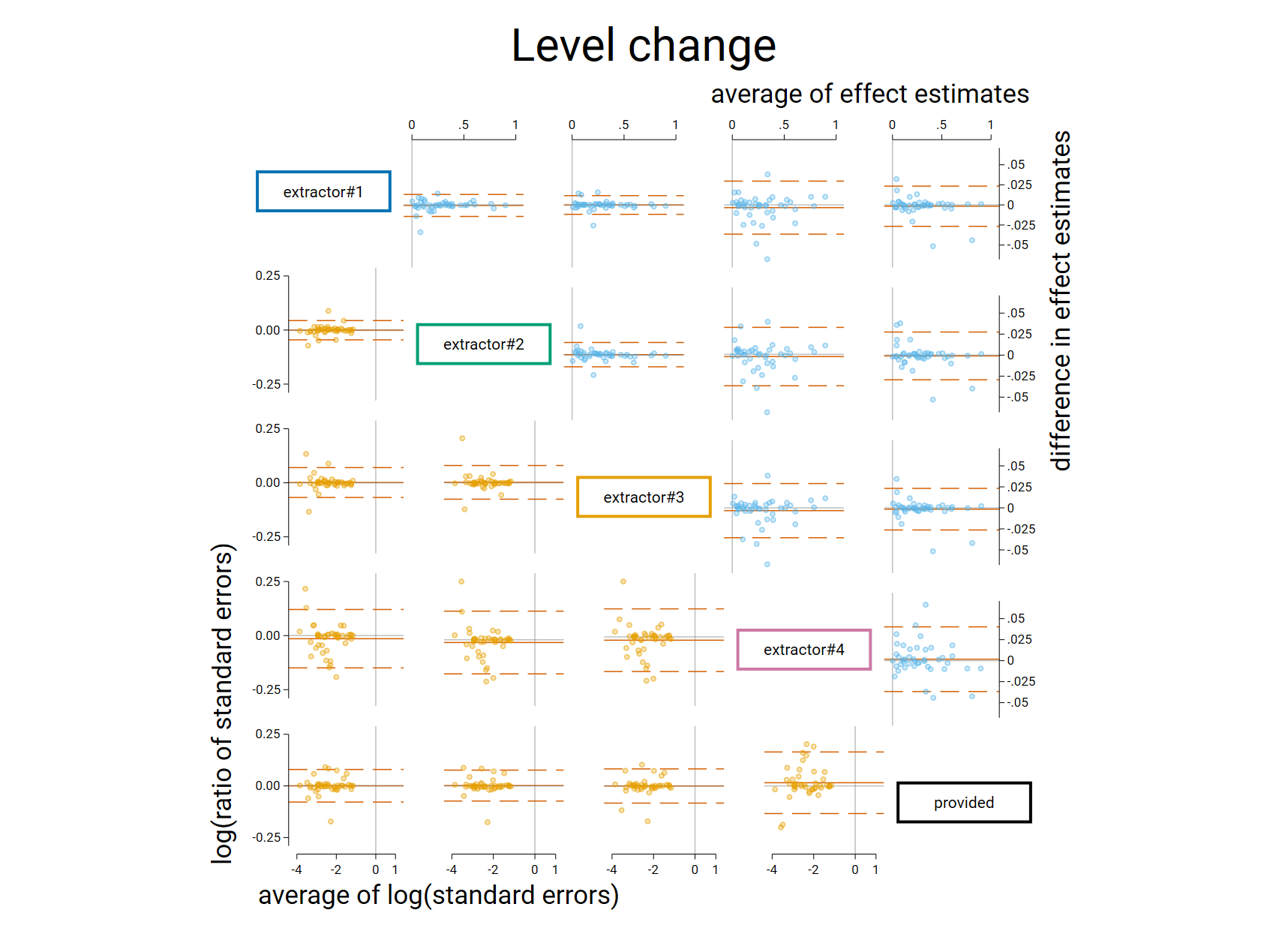


Figure S2.8: Bland Altman plot of standardised level change. Plots in the top triangle (blue points) show the difference in point estimates (row data source – column data source) on the vertical axis and average of the parameter estimates on the horizontal axis. Plots in the bottom triangle (orange points) show differences in standard errors on the vertical axis (= log(ratio of standard errors)) (column data source – row data source) and the average of the log of the standard errors on the horizontal axis. Red horizontal lines depict the average, red dashed lines depict the 95% limits of agreement (calculated as the average ±1.96*standard deviation of the differences). Grey lines indicate zero.

## Supplementary image 9: full size version of Figure 4


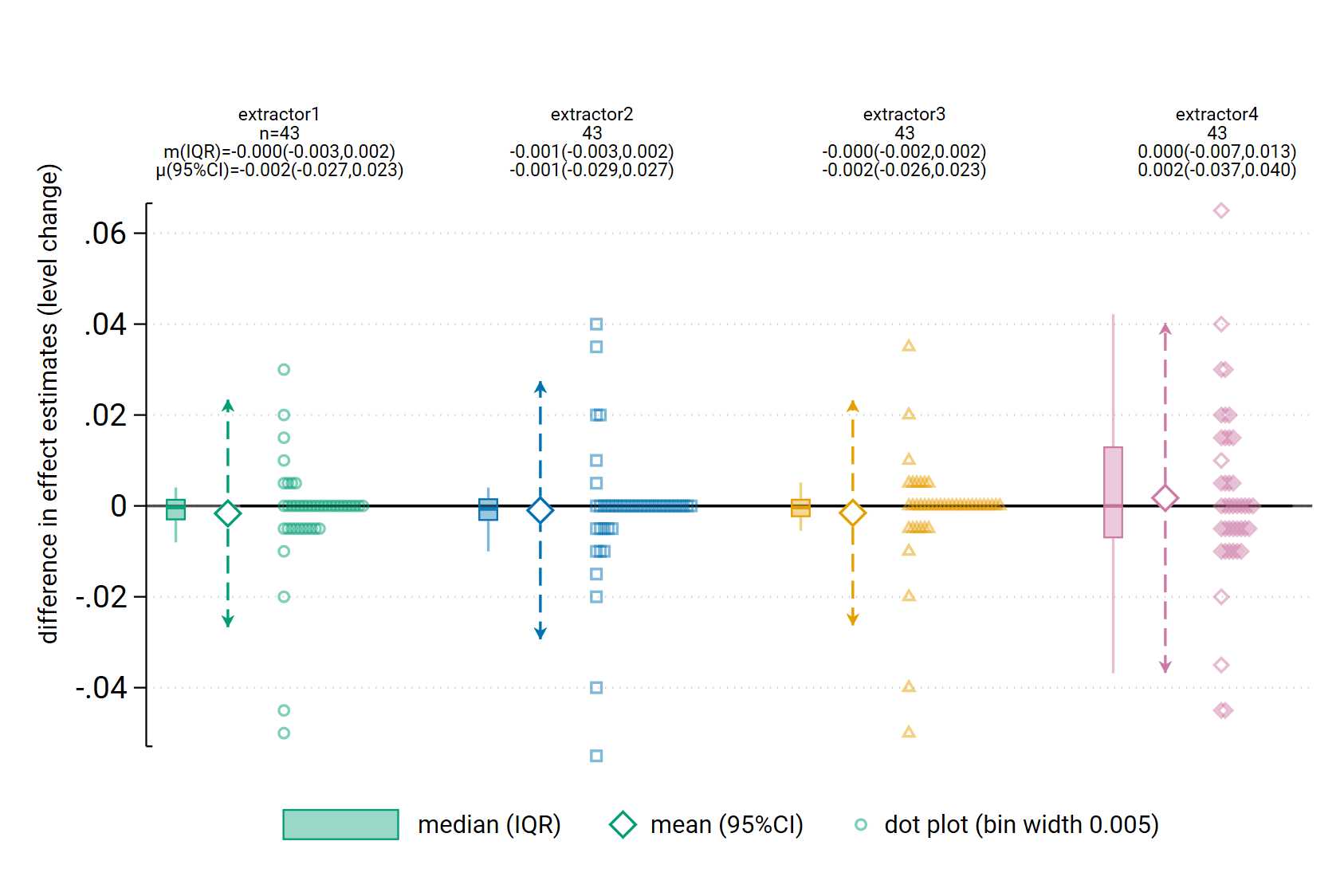


Figure S2.9: Dot plot showing difference in level change point estimates between extractor and provided data. Dot plot data has been aggregated to the nearest 0.005. Box plots show the median (m) (solid horizontal line), interquartile range (box) and lower and upper adjacent values (vertical lines). Large diamonds show the mean ($\mu$) with 95% limits of agreement (dashed arrows).

## Supplementary image 10: full size version of Figure 5


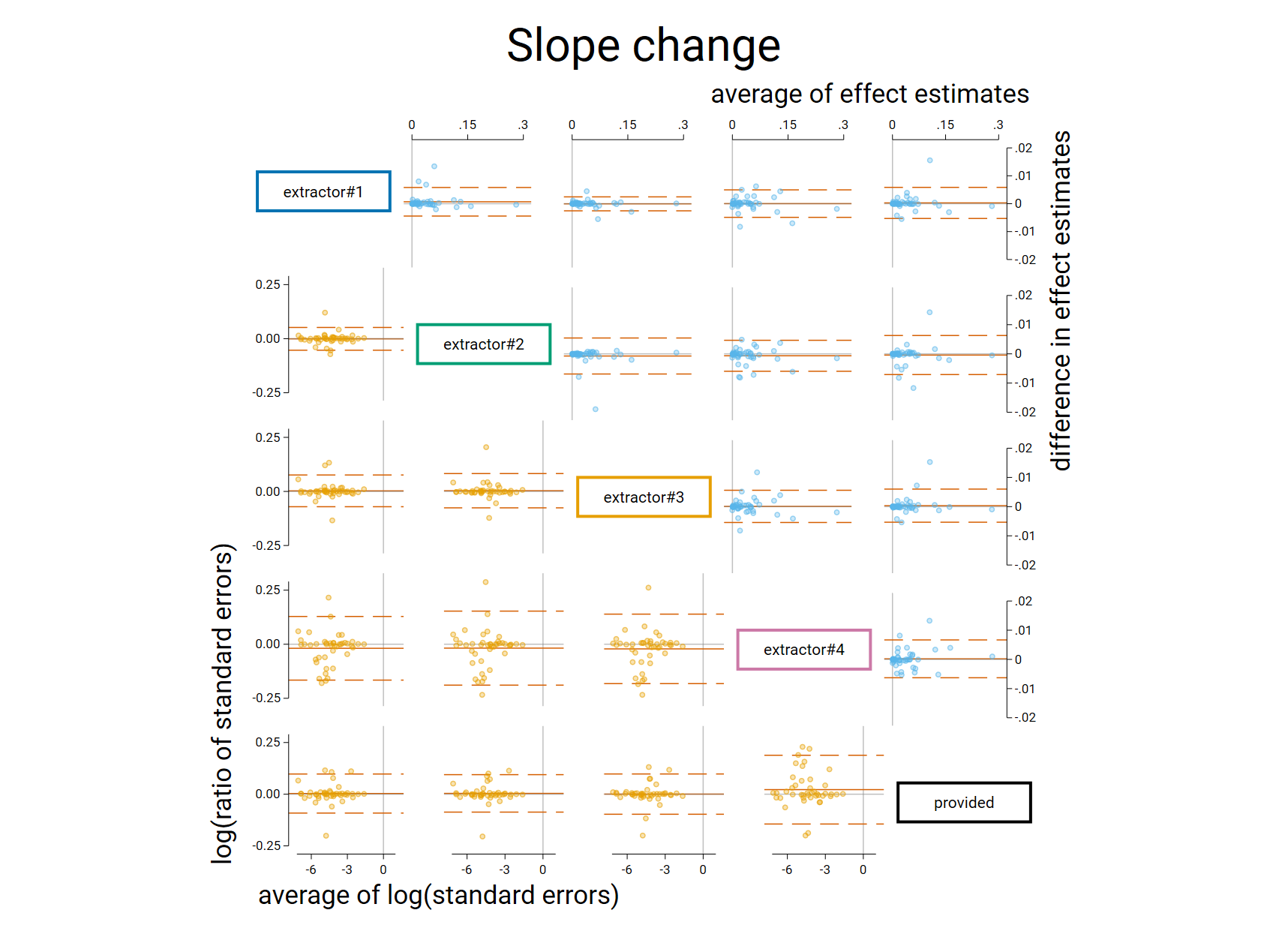


Figure S2.10: Bland Altman plot of standardised slope change. Plots in the top triangle (blue points) show the difference in point estimates (row data source – column data source) on the vertical axis and average of the parameter estimates on the horizontal axis. Plots in the bottom triangle (orange points) show differences in standard errors on the vertical axis (= log(ratio of standard errors)) (column data source – row data source) and the average of the log of the standard errors on the horizontal axis. Red horizontal lines depict the average, red dashed lines depict the 95% limits of agreement (calculated as the average ±1.96*standard deviation of the differences). Grey lines indicate zero.

## Supplementary image 11: full size version of Figure 6


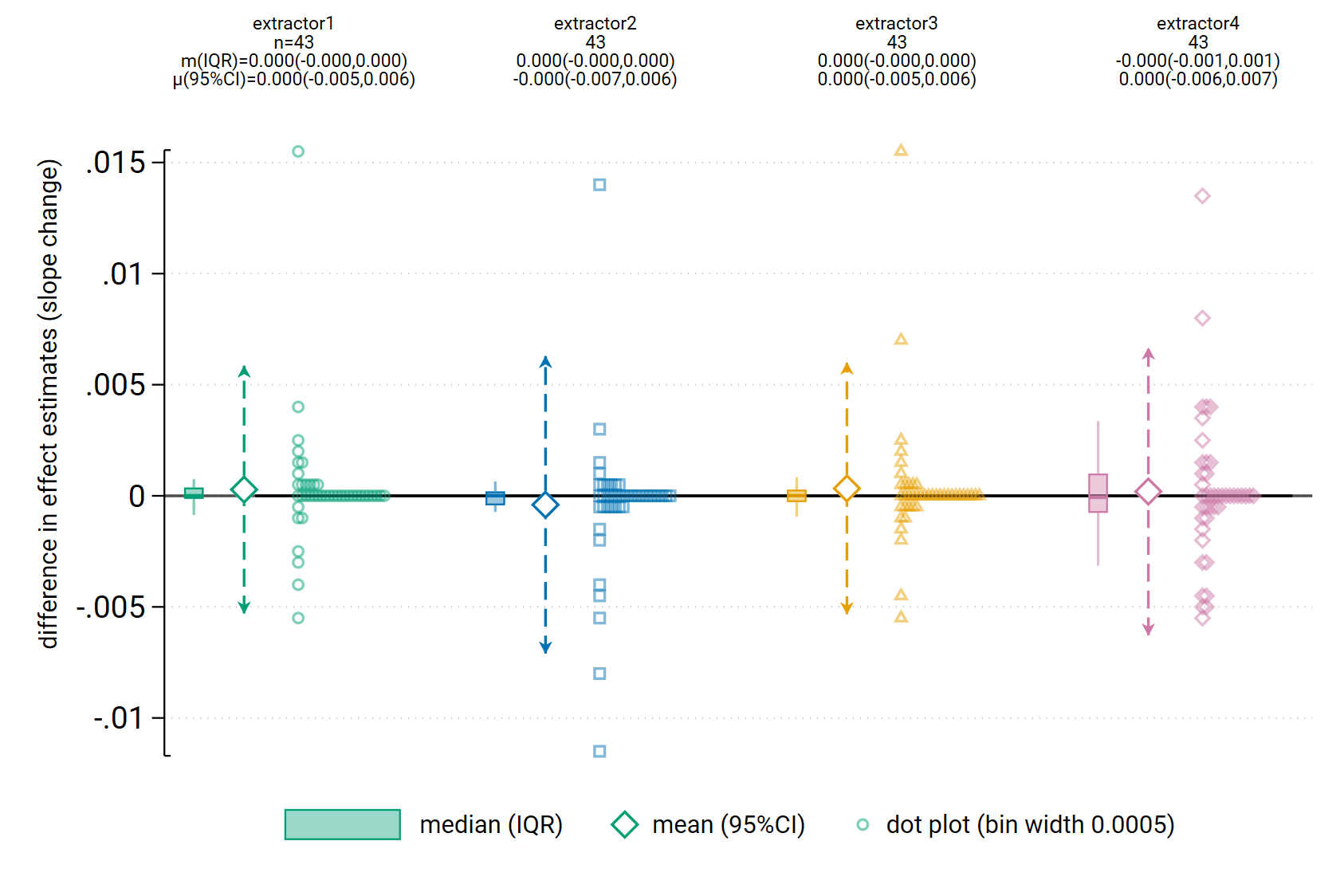


Figure S2.11: Dot plot showing difference in slope change point estimates between extractor and provided data. Dot plot data has been aggregated to the nearest 0.005. Box plots show the median (m) (solid horizontal line), interquartile range (box) and lower and upper adjacent values (vertical lines). Large diamonds show the mean ($\mu$) with 95% limits of agreement (dashed arrows).

## Supplementary image 12: full size version of Figure 7
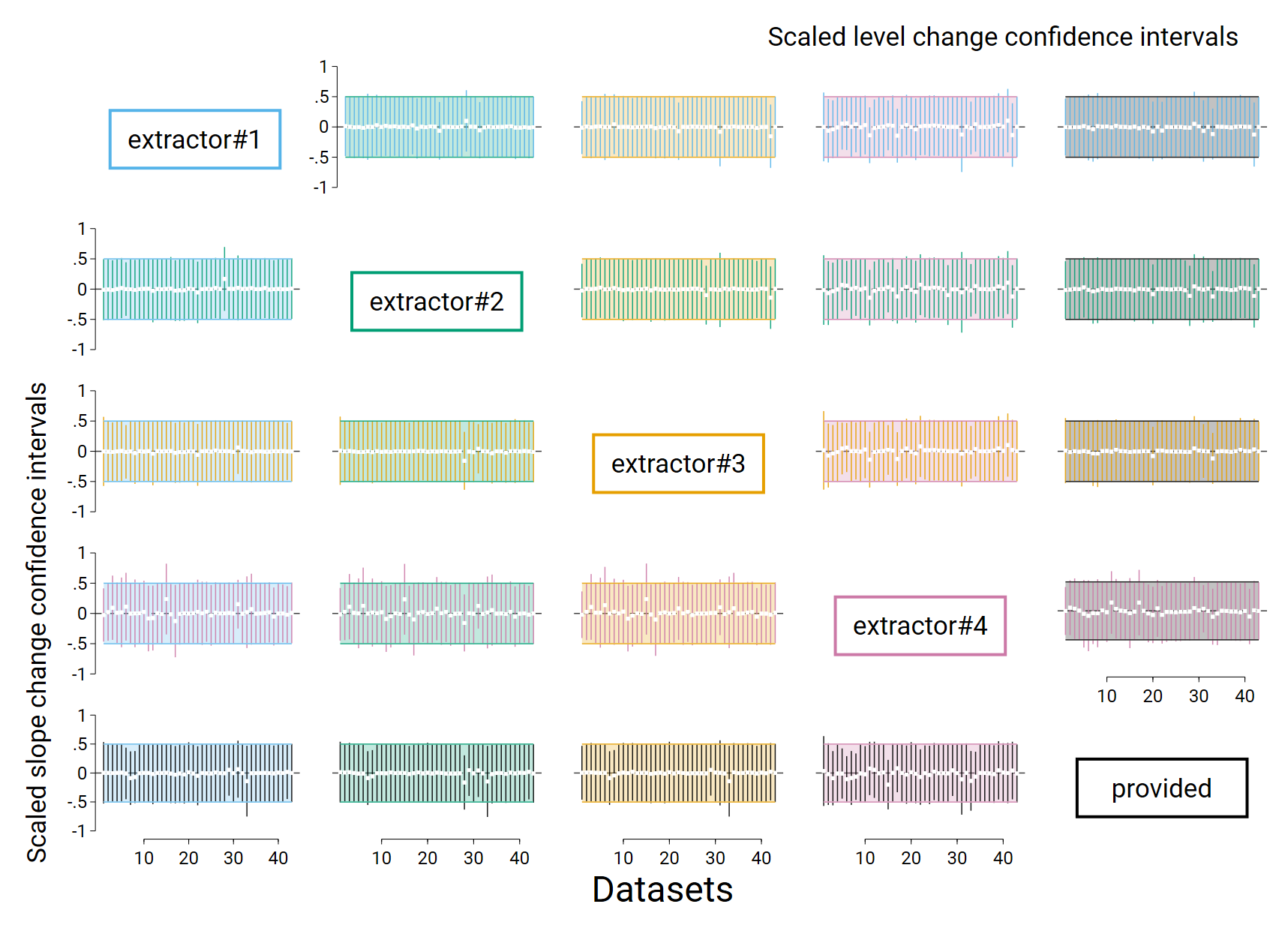


1. Figure S2.12: Pairwise confidence interval comparisons for immediate level change (top right triangle) and slope change (bottom left triangle). Each plot displays the 43 confidence intervals (CIs) (depicted as vertical lines), with each scaled so that the confidence interval from the reference data source spans -0.5 to 0.5 (shaded area). The reference method is the column data source (e.g. the plot in the second row, fifth column shows extractor 2 level change CIs (green) compared to provided (black)). Vertical lines falling entirely within the shaded area have smaller confidence intervals than the comparison, while lines extending beyond the shaded area have larger confidence intervals than the comparison. White dots indicate the point estimates.

## Supplementary material: original review methods

## Review methods

A detailed protocol for this study has been published (1).

### Eligibility criteria

Our definition of an ITS design was informed by the design features taxonomy for quasi-experimental designs (2, 3). ITS studies meeting the following criteria were included: 1) there were at least two segments separated by a clearly defined intervention or exposure with at least three points in each segment; 2) observations were collected on a group of individuals (e.g. community, hospital) at each time point; and 3) the study investigated the impact of a public health intervention or exposure that has public health implications. ITS studies meeting any of the following criteria were excluded: 1) written in a language other than English; 2) single-case design; or 3) were reported in a methodological paper examining ITS studies.

### Literature search

The bibliographic database PubMed was searched (to December 12^th^ 2017) using terms informed from previous search strategies developed to locate ITS studies (4, 5), terms used to describe the design in the methods section of published ITS studies (e.g. (6-8)), and controlled vocabulary (see original study (1), Additional file 2).

### Sample size and study selection

We included a sample of 200 ITS studies, which allowed estimation of the percentage of ITS studies with a particular element to within a maximum margin of error of 7% (assuming a prevalence of 50%). We included studies published between 2013 and 2017. Studies were stratified by year, and within each year, were randomly sampled until 40 were identified that met the inclusion criteria. We had planned to sample earlier years if we did not meet the target sample for any particular year, but this was not required.

Four authors (SLT, ABF, AK, JEM) piloted the eligibility criteria on 50 abstracts. A further 450 abstracts were screened by two authors (SLT and one of ABF, AK or JEM). The remaining abstracts (717) were screened by one author (SLT) and checked with at least two authors (ABF, AK, JEM) when there was uncertainty about any of the inclusion criteria and in all cases where inclusion criterion three was met (i.e. whether the study investigated the impact of a public health intervention or exposure that had public health implications). This latter check was undertaken since deciding on whether the study investigated a public health interruption was a subjective criterion to apply.

Four authors (SLT, ABF, AK, JEM) piloted the eligibility criteria on 10 full text articles. Two authors (SLT, AK) screened 44 full text articles, and the remaining articles were screened by one author (SLT) until 200 ITS studies were identified.

### Outcome(s) selection

For each included ITS study, multiple outcomes were potentially eligible for inclusion. We developed the following hierarchy, *a priori*, to select an outcome within each category of outcome type (binary, continuous, count, proportion): 1) ITS data availability – outcomes with data available to be extracted (either from tables or figures) were selected ahead of those without data; 2) stated primary outcome (or reported in the title or objectives); 3) first reported result outcome in the abstract; or, 4) first reported outcome in the results.

Other forms of multiplicity of time series arose that we had not considered *a priori*, and for which we developed selection rules *post hoc*. These included: 1) selection of the time series with the interruption in studies with a control group, and 2) selection of the first reported subgroup time series in studies with multiple subgroups.

Uncertainty in the selection of the review time series and outcomes were discussed by four authors (SLT, ABF, AK, JEM).

### References

1. Turner SL, Karahalios A, Forbes AB, Taljaard M, Grimshaw JM, Cheng AC, et al. Design characteristics and statistical methods used in interrupted time series studies evaluating public health interventions: protocol for a review. BMJ Open. 2019;9(1):e024096.

2. Reeves BC, Deeks JJ, Higgins JPT, Shea B, Tugwell P, Wells GA. Cochrane Handbook for Systematic Reviews of Interventions version 6.3. 2022 [cited May 2022]. In: Chapter 24: Including non-randomized studies on intervention effects [Internet]. Cochrane. 6.3. [cited May 2022].

3. Reeves BC, Wells GA, Waddington H. Quasi-experimental study designs series—paper 5: a checklist for classifying studies evaluating the effects on health interventions—a taxonomy without labels. Journal of Clinical Epidemiology. 2017;89:30-42.

4. Ramsay CR, Matowe L, Grilli R, Grimshaw JM, Thomas RE. Interrupted time series designs in health technology assessment: lessons from two systematic reviews of behavior change strategies. International Journal of Technology Assessment in Health Care. 2003;19(4):613-23.

5. Ewusie JE, Blondal E, Soobiah C, Beyene J, Thabane L, Straus SE, et al. Methods, applications, interpretations and challenges of interrupted time series (ITS) data: protocol for a scoping review. BMJ Open. 2017;7(6):e016018.

6. Cheng J, Benassi P, de Oliveira C, Zaheer J, Collins M, Kurdyak P. Impact of a mass media mental health campaign on psychiatric emergency department visits. Canadian Journal of Public Health. 2016;107(3):e303-e11.

7. Baker JM, Alonso WJ. Rotavirus vaccination takes seasonal signature of childhood diarrhea back to pre-sanitation era in Brazil. Journal of Infection. 2018;76(1):68-77.

8. Milojevic A, Armstrong B, Hashizume M, McAllister K, Faruque A, Yunus M, et al. Health Effects of Flooding in Rural Bangladesh. Epidemiology. 2012;23(1):107-15.
